# Comparative genomic analysis of *Streptomyces rapamycinicus* NRRL 5491 and its mutant overproducing rapamycin

**DOI:** 10.1038/s41598-022-14199-6

**Published:** 2022-06-18

**Authors:** Hee-Geun Jo, Joshua Julio Adidjaja, Do-Kyung Kim, Bu-Soo Park, Namil Lee, Byung-Kwan Cho, Hyun Uk Kim, Min-Kyu Oh

**Affiliations:** 1 Department of Chemical and Biological Engineering, Korea University, Seoul, 02841 Republic of Korea; 2 Department of Chemical and Biomolecular Engineering (BK21 Four), Korea Advanced Institute of Science and Technology (KAIST), Daejeon, 34141 Republic of Korea; 3 Department of Biological Sciences, Korea Advanced Institute of Science and Technology (KAIST), Daejeon, 34141 Republic of Korea

**Keywords:** Genomics, Industrial microbiology, Systems biology

## Abstract

*Streptomyces rapamycinicus* NRRL 5491 is a well-known producer of rapamycin, a secondary metabolite with useful bioactivities, including antifungal, antitumor, and immunosuppressive functions. For the enhanced rapamycin production, a rapamycin-overproducing strain SRMK07 was previously obtained as a result of random mutagenesis. To identify genomic changes that allowed the SRMK07 strain’s enhanced rapamycin production, genomes of the NRRL 5491 and SRMK07 strains were newly sequenced in this study. The resulting genome sequences of the wild-type and SRMK07 strains showed the size of 12.47 Mbp and 9.56 Mbp, respectively. Large deletions were observed at both end regions of the SRMK07 strain’s genome, which cover 17 biosynthetic gene clusters (BGCs) encoding secondary metabolites. Also, genes in a genomic region containing the rapamycin BGC were shown to be duplicated. Finally, comparative metabolic network analysis using these two strains’ genome-scale metabolic models revealed biochemical reactions with different metabolic fluxes, which were all associated with NADPH generation. Taken together, the genomic and computational approaches undertaken in this study suggest biological clues for the enhanced rapamycin production of the SRMK07 strain. These clues can also serve as a basis for systematic engineering of a production host for further enhanced rapamycin production.

## Introduction

*Streptomyces*, a representative genus of Gram-positive *Actinobacteria*, often contain 20–40 biosynthetic gene clusters (BGCs) in their genomes, and each BGC, upon proper expression, encodes specific secondary metabolites with a wide range of bioactivities including antibacterial, antifungal, antitumor, and immunosuppressive agents^1–5^. Therefore, *Streptomyces* species have been considered an important source of useful natural products, and have been the target of many metabolic engineering efforts^6–10^. However, some challenges exist in rational engineering of *Streptomyces* due to their GC-rich genomes and complex regulatory systems^11,12^. For these reasons, random mutagenesis is still conducted for the strain development of *Streptomyces* in addition to rational engineering^13–15^. Random mutagenesis approaches commonly use ultraviolet (UV) or chemical-based methods, but atmospheric and room-temperature plasma has also recently been utilized^15–18^. However, random mutagenesis always requires comprehensive subsequent genomic analysis to identify gene mutations that lead to the increased production performance of a production host.

In this study, we performed a comparative genomic analysis of the natural rapamycin producer *Streptomyces rapamycinicus* (formerly, *Streptomyces hygroscopicus*) NRRL 5491 (also ATCC 29253), and its mutant strain SRMK07 that overproduces rapamycin. Rapamycin is a hybrid of non-ribosomal peptide and polyketide with various useful bioactivities, including antifungal, antitumor, and immunosuppressive activities^19–23^. The biosynthesis of rapamycin requires 4,5-dihydroxycyclohex-1-enecarboxylic acid (DHCHC) as a starter unit, and also additional precursors, including malonyl-CoA, methylmalonyl-CoA, pipecolate, and NADPH. The SRMK07 strain was previously generated in our group via UV-based random mutagenesis^24^ (“Methods” section). Here, genomes of the wild-type and the SRMK07 strain were sequenced and compared in order to identify genomic changes in the SRMK07 strain that might be responsible for the enhanced rapamycin production. On the basis of the whole genome sequences, genome-scale metabolic models (GEMs) for these two strains were also reconstructed for metabolic analysis. This comparative genomic analysis revealed noteworthy differences that have likely contributed to the enhanced rapamycin production of the SRMK07 strain.

## Results

### Rapamycin production and growth of the SRMK07 strain

**Figure 1:**
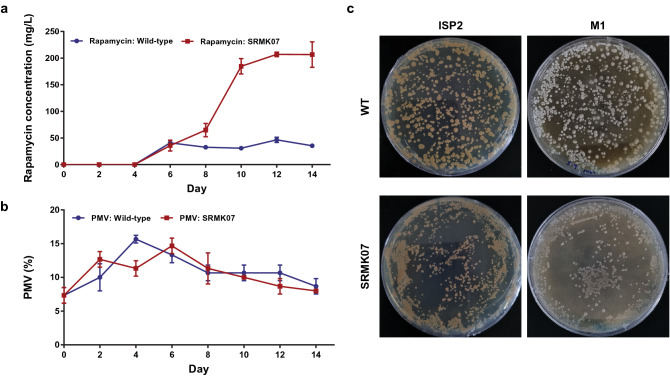
Rapamycin production performance and growth of *S. rapamycinicus* NRRL 5491 (wild-type) and its rapamycin-overproducing mutant SRMK07. (**a**,**b**) Rapamycin production performance (**a**) and growth (**b**) of the two strains. The presented data represent the mean from triplicate experiments, and the error bars indicate standard deviations. (**c**) Growth phenotypes of the two strains grown on ISP2 and M1 plates. The wild-type appeared to sporulate on the M1 plate, while the SRMK07 strain did not show any indication of sporulation. Images were taken on the seventh day of cultivation.

The rapamycin-overproducing mutant, *S. rapamycinicus* SRMK07, produced approximately 207 mg/L rapamycin, or around fourfold more rapamycin than the NRRL 5491 strain (Fig. 1a). Despite the significant improvement in the rapamycin production, the SRMK07 strain showed almost normal growth in comparison to the wild-type (Fig. 1b). The wild-type and the SRMK07 strain reached the peak of biomass accumulation on the fourth and sixth day, according to packed mycelium volume (PMV), respectively. Additionally, both strains were also grown on ISP2 and M1 plates to examine the morphology of their colonies and sporulation patterns, respectively (Fig. 1c and “Methods” section). Both strains grew well on ISP2 plates, but the wild-type colonies were greater in size; both strains did not sporulate on ISP2 plates. Meanwhile, on M1 plates, the wild-type formed spores, while the SRMK07 strain did not show any indication of sporulation.

### Whole genome sequencing of *S. rapamycinicus* NRRL 5491 and the SRMK07 strain

**Figure 2:**
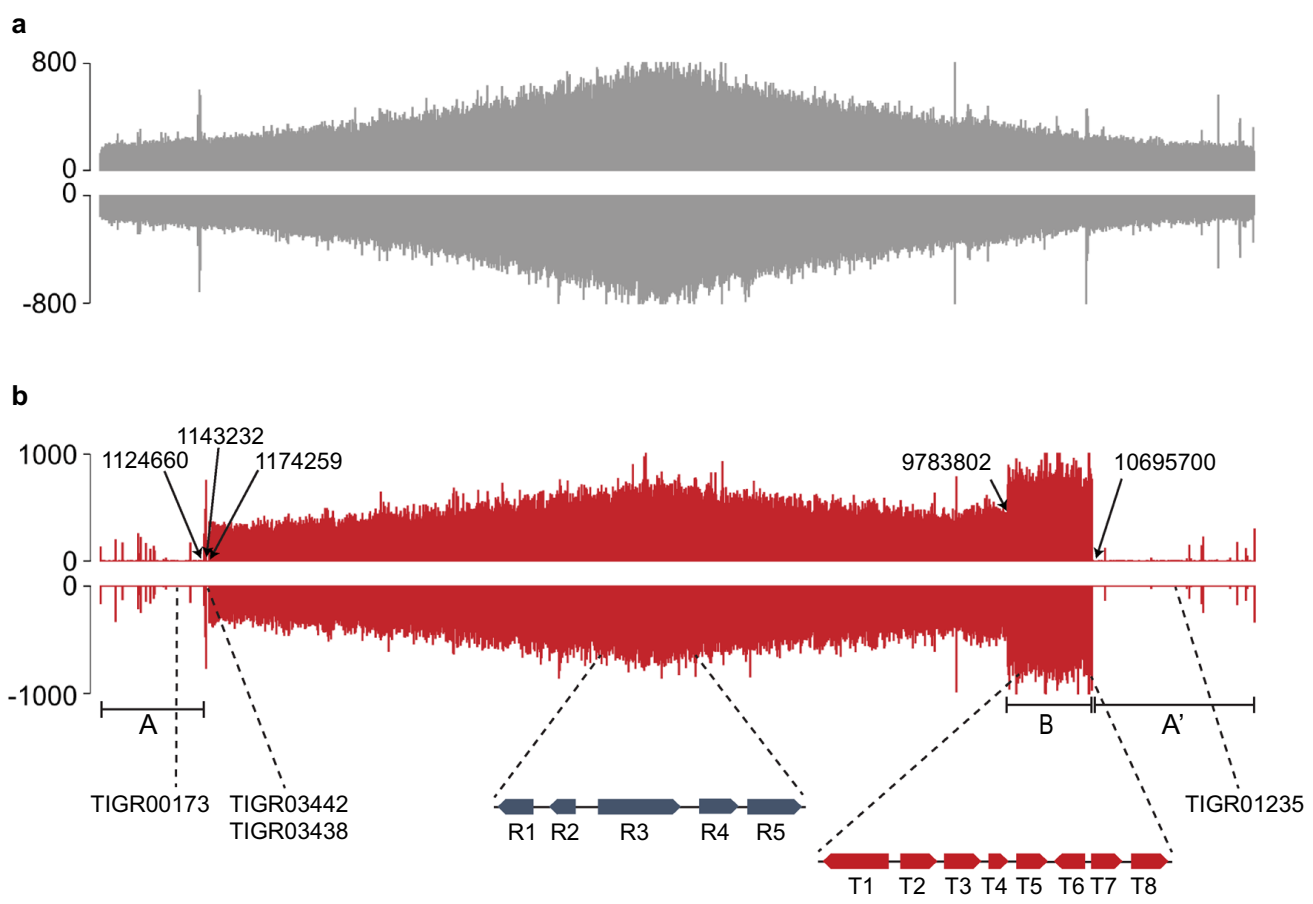
Profiles of Illumina reads from *S. rapamycinicus* NRRL 5491 (wild-type) and the SRMK07 strain, mapped on the wild-type’s genome assembled in this study. (**a**) Profile of Illumina reads from the wild-type. (**b**) Profile of Illumina reads from the SRMK07 strain. The data were visualized using SignalMap (Roche NimbleGen, Inc., Pleasanton, CA). ‘A’ and ‘A’’ indicate the potentially deleted regions, and ‘B’ indicates the potentially duplicated region in the SRMK07 strain’s genome. Information with dashed lines correspond to the location of target genes for the relative quantification analysis using qPCR (Fig. 3) as well as the deleted core genes (Table 2) in the SRMK07 strain’s genome.

We next conducted whole genome sequencing (WGS) of the NRRL 5491 and SRMK07 strains, using both PacBio and Illumina platforms, to identify genomic changes in the SRMK07 strain that have led to its high production performance of rapamycin. The resulting whole genome sequences of the wild-type and the SRMK07 appeared to be 12.47 Mbp and 9.56 Mbp, respectively (Fig. 2). In contrast to the SRMK07 strain’s genome that was initially obtained as a single contig, the wild-type’s genome data were obtained as seven contigs. To resolve this problem, two independent sequences of *S. rapamycinicus* NRRL 5491 genome, currently available in the NCBI database (GCA_003675955.1^25^ and GCA_000418455.1^26^), were used as references to connect the seven contigs of our wild-type’s genome. Among these two sequences, only GCA_000418455.1 is represented as a single contig although it contains multiple sequencing gaps. In contrast, the second sequence (i.e., GCA_003675955.1) contains four contigs, but fortunately, lacks sequencing gaps. Therefore, we utilized GCA_000418455.1 as an initial framework to determine the correct order of our own NRRL 5491 seven contigs, while GCA_003675955.1 served as a template to fill any sequencing gaps. The assembled wild-type genome sequence showed a size (12.47 Mbp) comparable to that of GCA_000418455.1 (12.70 Mbp).

### Comparative genomic analysis based on Illumina reads mapping

Comparison of the genomes of the wild-type and the SRMK07 strain showed a difference of about 2.91 Mbp; 10,140 protein-coding genes were predicted from the wild-type genome, while only 7757 protein-coding genes were predicted from the SRMK07 genome (Fig. 2), which indicates large genomic deletions in the SRMK07 genome as a result of the random mutagenesis. To further analyze the genomic differences between the wild-type and the SRMK07 strain, Illumina sequencing reads of the SRMK07 strain were mapped on the assembled wild-type genome (Fig. 2). As a result, large deletions were observed at both end regions of the SRMK07 genome, which corresponded to approximately 2.91 Mbp (the regions ‘A’ and ‘A’’ in Fig. 2b); this deletion size was almost consistent with the difference in the genome sizes of the wild-type and the SRMK07 strain.

These genomic deletions in the SRMK07 strain were further confirmed by PCR experiments (Supplementary Fig. S1, Supplementary Table S1) and comparative genomic analysis (Supplementary Data 1). For the PCR experiments, primers were designed to target genes located in either end region of the wild-type’s genome, which were expected to be absent in the SRMK07 strain’s genome. Indeed, as a result of the PCR, target bands with expected size were obtained only from the wild-type’s genomic DNA (gDNA), and not from the SRMK07 strain’s genome (Supplementary Fig. S1). However, further thorough analysis will be necessary to confirm these potential genomic deletions in the SRMK07 strain because *Streptomyces* species have a linear chromosome with both ends having terminal inverted repeats (TIRs), and these TIRs make firm mapping of the borders of the deletions difficult. Full information on conflict positions in nucleotide sequences as well as missing genes in the SRMK07 strain in comparison with the wild-type is available in Supplementary Data 1.

We subsequently examined whether secondary metabolite BGCs in the SRMK07 strain were affected by the genomic deletions by running antiSMASH 5.0 for genomes of the wild-type and the SRMK07 strain^27^. As a result, the wild-type was predicted to have 52 BGCs, whereas 17 BGCs appeared to be lost in the SRMK07 strain (Table 1). Since nine of these missing BGCs encode polyketides or hybrids of non-ribosomal peptides and polyketides, the loss of these BGCs in the SRMK07 strain might have enhanced the production of rapamycin by redirecting precursors necessary for the rapamycin biosynthesis.

**Table 1:** Biosynthetic gene clusters (BGCs) that appeared to be absent in the SRMK07 strain’s genome.

Location in the NRRL 5491 genome (bp)^a^	Secondary metabolite encoded by a predicted BGC^b^
3–106,876	Lobophorin A
491,617–540,884	Coelichelin
859,144–868,059	Cyphomycin
1,024,465–1,112,648	Azalomycin F3a
10,679,301–10,730,137	Atratumycin
10,976,611–11,092,238	Meridamycin
11,099,441–11,181,894	Hygrocin A/B
11,336,124–11,459,137	Bafilomycin B1
11,461,501–11,471,719	Bacteriocin
11,477,329–11,554,061	Dechlorocuracomycin
11,606,908–11,627,298	Brasilicardin A
11,765,022–11,785,930	Terpene
11,835,848–11,877,612	Echoside A, B, C, D and E
11,969,269–11,998,132	Sch-47554 and Sch-47555
12,035,215–12,099,769	Herboxidiene
12,222,724–12,276,054	NRPS^c^
12,289,607–12,332,240	Geldanamycin

^a^BGCs were detected using antiSMASH version 5.0^27^.

^b^Metabolites in red are polyketides or hybrids of non-ribosomal peptide and polyketide.

^c^Non-ribosomal peptide synthetase.

**Figure 3:**
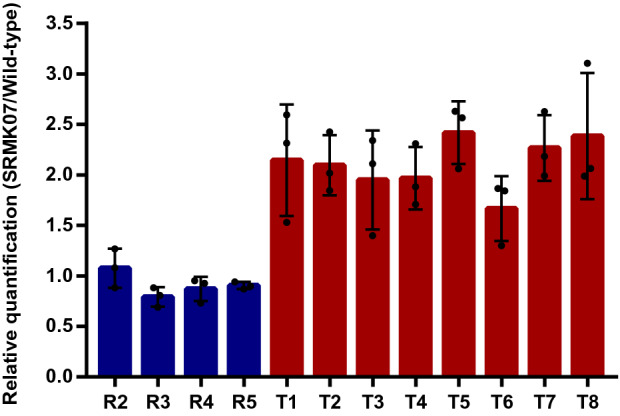
Relative quantification of genes from the potentially duplicated region in the SRMK07 strain’s genome. For this, five reference single-copy genes (‘R1’ to ‘R5’ defined below) and eight genes (‘T1’ to ‘T8’ defined below) from the potentially duplicated region in the SRMK07 strain’s genome were first selected for the qPCR experiments. A gene ‘R1’ encoding NADH-quinone oxidoreductase subunit H (5,392,720**–**5,393,685 bp in the NRRL 5491 genome) was used as a control to measure the relative quantity of the other four single-copy genes (blue bars), and the eight genes from the potentially duplicated region (red bars). Genes ‘R2’, ‘R3’, ‘R4’, and ‘R5’ are those known to exist as a single copy across *Streptomyces* species, and encode the following proteins, respectively (along with the chromosome location in the NRRL 5491 genome): RtcB family protein (5,488,049–5,488,159 bp); RNA helicase (5,756,656–5,760,582 bp); aspartate kinase (6,341,319–6,342,599 bp); and type I DNA topoisomerase (6,399,363–6,402,227 bp). Genes ‘T1’, ‘T2’, ‘T3’, ‘T4’, ‘T5’, ‘T6’, ‘T7’, and ‘T8’ from the potentially duplicated region encode the following proteins, respectively: 3-ketoacyl-CoA thiolase (9,778,772–9,809,242 bp); regulatory protein AfsR (9,875,692–9,877,524 bp); l-lysine cyclodeaminase (9,900,735–9,901,766 bp); ferredoxin (9,904,048–9,904,284 bp); glycerol uptake operon antiterminator regulatory protein (10,001,392–10,002,039 bp); hypothetical protein (10,003,629–10,004,255 bp); SDR family oxidoreductase (10,035,843–10,036,631 bp); and putative ABC transporter ATP-binding protein YbiT (10,693,310–10,694,929 bp). ‘T1’ and ‘T8’ represent the start and end regions of the potentially duplicated region. ‘T3’, ‘T4’ and ‘T6’ belong to rapamycin BGC. The primers used for these qPCR experiments are available in Supplementary Table S2. The presented data represent the mean from triplicate experiments, and the error bars indicate standard deviations.

Interestingly, a genomic region (9,783,802–10,695,700 bp in the wild-type genome) was observed in the SRMK07 strain where the number of the mapped reads was notably greater than other regions of the genome (1,174,259–9,783,802 bp in the wild-type genome) by approximately twofold (the region ‘B’ in Fig. 2b); this genomic region strongly indicates the duplication of genes. Since this genomic region in the SRMK07 strain covers the rapamycin BGC (8,583,793–8,830,075 bp in the SRMK07 genome, which corresponds to 9,758,976–10,004,25 bp in the wild-type genome), duplication of genes in this region might have also contributed to the enhanced rapamycin production. To further verify the duplication of this genomic region, we performed real-time PCR (qPCR) for relative quantification of genes from the potentially duplicated region in comparison with genes from other regions that are known to exist as a single copy across various *Streptomyces* species (Fig. 3, Supplementary Table S2); information on single-copy genes was obtained from OrthoDB^28^. For this analysis, five reference single-copy genes were selected, which encode: NADH-quinone oxidoreductase subunit H; RtcB family protein; RNA helicase; aspartate kinase; and type I DNA topoisomerase. Likewise, eight genes were selected from the potentially duplicated region in the SRMK07 strain’s genome, which encode: 3-ketoacyl-CoA thiolase; regulatory protein AfsR; l-lysine cyclodeaminase; ferredoxin; glycerol uptake operon antiterminator regulatory protein; a hypothetical protein encoded by a rapamycin biosynthetic gene; SDR family oxidoreductase; and putative ABC transporter ATP-binding protein YbiT. l-lysine cyclodeaminase (encoded by *rapL*), ferredoxin (encoded by *rapO* gene), and the hypothetical protein all belong to the rapamycin BGC. The qPCR results with the gene encoding NADH-quinone oxidoreductase subunit H as a control showed that the relative quantities of the reference single-copy genes in the SRMK07 genome ranged from 0.7 to 1.3, while the relative quantities of the genes from the potentially duplicated region were close to 2 (Fig. 3). These results strongly suggest the duplication of the genomic region in the SRMK07 strain where the rapamycin BGC is located.

### Core gene analysis

The SRMK07 strain showed the normal growth despite the large genomic deletions. This observation raised a question on the presence of core genes in this strain that are necessary for the normal growth; the core genes here refer to those present in genomes of the vast majority of biologically related organisms, for example, *Streptomyces* species in this study, likely because of the biological importance^29,30^. To examine the distribution of core genes in the SRMK07 strain’s genome, a software program ‘Antibiotic Resistant Target Seeker’ (ARTS) was used, which allows the detection of core genes, including housekeeping genes and resistance genes associated with BGCs^31^. As a result, ARTS predicted 393 and 389 core genes (out of 10,140 and 7757 protein-coding genes, respectively) from genomes of the wild-type and the SRMK07 strain, respectively (Table 2, Supplementary Data 2). Hence, only four core genes were predicted to be missing in the SRMK07 strain. These four genes include TIGR01235, TIGR03442, TIGR03438, and TIGR00173 (all TIGRFAM identifiers^32^), which encode: pyruvate carboxylase; ergothioneine biosynthesis protein EgtC (or γ-glutamyl-hercynylcysteine sulfoxide encoded); ergothioneine-biosynthetic methyltransferase EgtD (or histidine N-alpha-methyltransferase); and 2-succinyl-5-enolpyruvyl-6-hydroxy-3-cyclohexene-1-carboxylate synthase MenD, respectively. It should be noted that evidence for the possible presence of paralogs of these four genes was not found in the SRMK07 genome according to ARTS and OrthoDB.

**Table 2:** Deleted core genes in the SRMK07 strain’s genome.

Core gene predicted by ARTS^a^	Location in the NRRL 5491 genome (bp)	Protein name	Duplication in wild-type^b^	Copy number in wild-type	Duplication in SRMK07	Copy number in SRMK07
TIGR01235	11,590,533–11,593,911	Pyruvate carboxylase	No	1	–	–
TIGR03442	1,160,018–1,160,774	Ergothioneine biosynthesis protein	No	1	–	–
TIGR03438	1,160,770–1,161,736	Ergothioneine-biosynthetic methyltransferase	No	1	–	–
TIGR00173	832,039–833,806	2-Succinyl-5-enolpyruvyl-6-hydroxy-3-cyclohexene-1-carboxylate synthase	No	1	–	–
TIGR01356	4,423,896–4,425,273 11,829,102–11,830,458	3-Phosphoshikimate 1-carboxyvinyltransferase	Yes	2	No	1
TIGR00753	2,012,607–2,013,480 10,469,006–10,469,843 11,556,234–11,557,071	Undecaprenyl-diphosphatase	Yes	3	Yes	2
TIGR01900	4,510,510–4,511,581 11,545,436–11,546,543	Succinyl-diaminopimelate desuccinylase	Yes	2	No	1
TIGR01311	1,818,956–1,820,474 9,256,421–9,257,969 10,360,222–10,361,722 10,637,461–10,638,994 11,557,789–11,559,295	Glycerol kinase	Yes	5	Yes	4
TIGR01312	1,144,115–1,145,573 10,338,941–10,340,453	Xylulokinase	Yes	2	No	1
TIGR01751	1,592,678–1,594,019 3,325,824–3,327,171 9,491,404–9,492,772 10,704,812–10,706,060	Crotonyl-CoA carboxylase/reductase	Yes	4	Yes	3
TIGR00119	4,045,336–4,045,861 11,950,151–11,950,664	Acetolactate synthase, small subunit	Yes	2	No	1
SHMT	539,567–540,884 4,104,899–4,106,180 4,264,217–4,265,507 6,883,294–6,884,566	Serine hydroxymethyltransferase	Yes	4	Yes	3
Ribosomal_S14	5,224,623–5,224,809 10,874,927–10,875,206	Ribosomal protein S14p/S29e	Yes	2	No	1
TIGR01915	8,473,740–8,474,514 9,199,776–9,200,484 10,826,539–10,827,214 12,188,951–12,189,509	NADPH-dependent F420 reductase	Yes	4	Yes	2

^a^Identifiers (IDs) of the detected core genes were obtained from ARTS, which are mostly TIGRFAM IDs^32^.

^b^‘Duplication’ indicates the presence of a gene with a greater copy number than the average copy number of this gene present in other organisms.

A close examination of metabolic genes in the SRMK07 strain suggested that the loss of these four core genes should not affect the overall metabolic activities of the SRMK07 strain. Pyruvate carboxylase (TIGR01235) is an anaplerotic enzyme, and is involved in regulating a phosphoenolpyruvate (PEP)-pyruvate-oxaloacetate pool that is critical for the optimal distribution of metabolic fluxes in central carbon metabolism^33^. Despite the loss of this gene, other genes involved in regulating the PEP-pyruvate-oxaloacetate pool were still present in the SRMK07, including PEP carboxykinase, PEP carboxylase, malic enzyme, and malate dehydrogenase. Next, EgtC (TIGR03442) and EgtD (TIGR03438) are involved in the biosynthesis of ergothioneine that detoxifies reactive oxygen species and reactive nitrogen species for redox homeostasis, and the absence of ergothioneine results in higher oxidative stress^34,35^. Biological roles of ergothioneine in Gram-positive bacteria can be complemented by other detoxifying molecules, such as mycothiol, and glutathione^34,35^. Our genomic analysis of the wild-type and the SRMK07 showed that both strains carry intact genes for the biosynthesis of mycothiol^36^ (i.e., *mshA*, *mshB*, *mshC*, and *mshD*) and glutathione. Finally, *menD* (TIGR00173) is part of the genes, *menABCDEFGH*, that encode the biosynthesis of menaquinone; menaquinone plays an important role in the electron transport in Gram-positive bacteria^37^. Fortunately, an alternative biosynthetic pathway for menaquinone, known as futalosine pathway, has also been reported in *Streptomyces coelicolor*^38–40^. Homologs of the genes in this futalosine pathway were found to be present in both the wild-type and the SRMK07 strain (Supplementary Table S3).

Taken together, the additional genes that were found intact in the SRMK07 strain have the possibility to complement the function of the absent four core genes (i.e., TIGR01235, TIGR03442, TIGR03438, and TIGR00173), and would allow the SRMK07 strain’s normal growth despite the large genomic deletions. Moreover, there were core genes that exist in multiple copies in the wild-type, but at least a single copy was found for all these core genes in the SRMK07 strain (Table 2).

### Comparative metabolic network analysis

**Figure 4:**
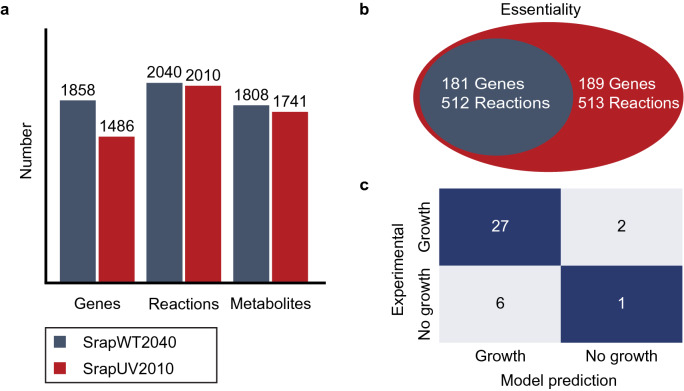
Statistics and simulation results of the genome-scale metabolic models (GEMs) of *S. rapamycinicus* NRRL 5491 (wild-type) and its mutant SRMK07. (**a**) Number of genes, reactions, and metabolites of the GEMs, SrapWT2040 and SrapUV2010, that represent the wild-type and its mutant SRMK07, respectively. (**b**) Number of essential genes and essential reactions predicted using SrapWT2040 and SrapUV2010. SrapUV2010 was predicted to have eight additional essential genes, and one additional essential reaction in comparison with SrapWT2040. (**c**) The growth prediction results using SrapWT2040 in comparison with the reported growth data that involved 17 individual carbon sources (Supplementary Table S4) and 19 individual nitrogen sources (Supplementary Table S5). It should be noted that SrapUV2010 also generated the same prediction accuracy as SrapWT2040.

Enhanced production of a secondary metabolite might also be linked with changes in a metabolic network, providing precursors and energy molecules necessary for rapamycin biosynthesis. To examine this question, we reconstructed GEMs, SrapWT2040 and SrapUV2010, that describe the metabolism of the NRRL 5491 and SRMK07 strains, respectively (Supplementary Data 3, 4). In contrast to 2383 protein-coding genes that appeared to be deleted in the SMRK07 strain as a result of the random mutagenesis, the constructed SrapUV2010 appeared to have only 30 fewer biochemical reactions than SrapWT2040; these 30 biochemical reactions are associated with a total of 372 metabolic genes (Fig. 4a, Table 3). As expected, biochemical reactions associated with a pathway ‘Biosynthesis of secondary metabolites’ were shown to be most affected in SrapUV2010; six corresponding biochemical reactions, out of the 30 reactions, were missing in SrapUV2010. Additional metabolic pathways that were affected by the random mutagenesis include: central carbon metabolism (i.e., fructose, mannose, glyoxylate, and TCA cycle), amino acid metabolism (i.e., phenylalanine, arginine, proline, and glycine), and degradation pathways (i.e., benzoate, styrene, and polycyclic aromatic hydrocarbon).

**Table 3:** List of 30 biochemical reactions available in SrapWT2040 (wild-type), but absent in SrapUV2010 (SRMK07 strain).

Metabolic pathway	Reaction ID	Protein name	Reaction^a^
Fructose and mannose metabolism	XYLI1	Xylose isomerase	xyl__D_c ⇔ xylu__D_c
Fructose and mannose metabolism	XYLI2	Xylose isomerase	glc__D_c ⇔ fru_c
Glyoxylate and dicarboxylate metabolism	TRSARr	Tartronate semialdehyde reductase	2h3oppan_c + h_c + nadh_c ⇔ glyc__R_c + nad_c
Glyoxylate and dicarboxylate metabolism	HPYRI	Hydroxypyruvate isomerase	hpyr_c ⇔ 2h3oppan_c
Carbohydrate acid metabolism	GLCRD	Glucarate dehydratase	glcr_c → 5dh4dglc_c + h2o_c
TCA cycle	FRD8	Fumarate reductase (menaquione-9)	fum_c + mql9_c → mqn9_c + succ_c
Phenylalanine metabolism	PHACTE	Phenylacetyl-CoA thioesterase	h2o_c + phaccoa_c → coa_c + h_c + pac_c
Sulfur metabolism	TAUDO	Taurine dioxygenase	akg_c + o2_c + taur_c → aacald_c + co2_c + h_c + so3_c + succ_c
Benzoate degradation	3OADPCOAT	3-Oxoadipate CoA-transferase	3oxoadp_c + succoa_c → oxadpcoa_c + succ_c
Arginine and proline metabolism	AGMDA	Agmatine deiminase	agm_c + h2o_c → cptrc_c + nh4_c
Arginine and proline metabolism	AGMT	Agmatinase	agm_c + h2o_c → ptrc_c + urea_c
Calcium-dependent antibiotic biosynthesis	CDAS12	Hexenoyl-CoA monooxygenase	h_c + hx2coa_c + nadph_c + o2_c → ephxcoa_c + h2o_c + nadp_c
Lipoate salvage I	LIPAMPL	Lipoyl-adenylate protein ligase	lipoamp_c → amp_c + h_c + lipopb_c
Lipoate salvage I	LIPATPT	Lipoate-ATP adenylate transferase	atp_c + lipoate_c → lipoamp_c + ppi_c
Lipoate metabolism	OCTNLL	Octanoate non-lipoylated apo domain ligase	atp_c + h_c + octa_c → amp_c + octapb_c + ppi_c
Glycine, serine and threonine metabolism	GLYAT	Glycine C-acetyltransferase	accoa_c + gly_c ⇔ 2aobut_c + coa_c
Ascorbate and aldarate metabolism	GLCRD2	d-glucarate hydro-lyase	glcr_c ⇔ 2dh3dglc_c + h2o_c
Styrene degradation	R05551	Acrylamidase	aa_c + h2o_c ⇔ acryl_c + nh4_c
Transport reaction	HOMt2	l-homoserineserine efflux via proton symport	h_e + hom__L_c → h_c + hom__L_e
Synthesis and degradation of ketone bodies; Valine, leucine and isoleucine degradation; Butanoate metabolism	OCOAT1	3-Oxoacid CoA-transferase (Succinyl-CoA: acetoacetate)	acac_c + succoa_c → aacoa_c + succ_c
Dioxin degradation; Polycyclic aromatic hydrocarbon degradation; Naphthalene degradation	SALOR	Salicylate 1-monooxygenase	2.0 h_c + nadh_c + o2_c + salc_c → catechol_c + co2_c + h2o_c + nad_c
Biosynthesis of secondary metabolites	ACTS16	actVA-5/actVB monooxygenase	ddhkACPact_c + o2_c → dhkACPact_c + h2o_c
Biosynthesis of secondary metabolites	ACTS17	R09312	dhkACPact_c + h2o_c + nadh_c → ACPact_c + dhkdhqn_c + nad_c
Biosynthesis of secondary metabolites	ACTS18	Dihydrokalafungin-dihydroquinone,FMNH2:oxygen oxidoreductase (hydroxylating)	dhkdhqn_c + fmnh2_c + o2_c ⇔ fmn_c + h2o_c + hdhk_c
Biosynthesis of secondary metabolites	ACTS16b	R09314	ddhkACPact_c + fmnh2_c + o2_c → dhkACPact_c + fmn_c + h2o_c + 2.0 h_c
Biosynthesis of secondary metabolites	R00918	Propanoyl-CoA:(2S)-methylmalonyl-CoA malonyltransferase (cyclizing);	MNXM2496_c + 6.0 co2_c + 7.0 coa_c + h2o_c + 6.0 nadp_c ⇔ 12.0 h_c + 6.0 mmcoa__S_c + 6.0 nadph_c + ppcoa_c
Biosynthesis of secondary metabolites	R02225	ATP:streptomycin 6-phosphotransferase	Stmyn_c + atp_c ⇔ MNXM2286_c + adp_c + h_c
Biosynthesis of secondary metabolites	R07253	Acyl-CoA:malonyl-CoA C-acyltransferase (decarboxylating, oxoacyl-reducing, thioester-hydrolysing and cyclizing)	MNXM2093_c + 3.0 co2_c + 4.0 coa_c + h2o_c + nadp_c ⇔ accoa_c + 3.0 h_c + 3.0 malcoa_c + nadph_c
	NODOx	Nitric oxide dioxygenase	nadh_c + 2.0 no_c + 2.0 o2_c → h_c + nad_c + 2.0 no3_c
	NODOy	Nitric oxide dioxygenase	nadph_c + 2.0 no_c + 2.0 o2_c → h_c + nadp_c + 2.0 no3_c

^a^Metabolite abbreviations: *2aobut_c*L-2-amino-3-oxobutanoate, *2dh3dglc_c* 2-dehydro-3-deoxy-D-glucarate, *2h3oppan_c* 2-hydroxy-3-oxopropanoate, *3oxoadp_c* 3-oxoadipate, *5dh4dglc_c*5-dehydro-4-deoxy-D-glucarate, *MNXM2093_c* 6-methylsalicylate, *MNXM2286_c*streptomycin 6-phosphate, *MNXM2496_c* 6-deoxyerythronolide B, *Stmyn_c* streptomycin, *aa_c* acrylamide, *aacald_c *aminoacetaldehyde,* aacoa_c* acetoacetyl-CoA,*acac_c* acetoacetate, *accoa_c* acetyl-CoA, *ACPact_c* acyl carrier protein (specific to actinorhodin PKS), *acryl_c* acrylate,*adp_c* ADP, *agm_c* agmatine, *akg_c* 2-oxoglutarate, *amp_c* AMP,*atp_c* ATP, catechol_c catechol, *co2_c* CO_2_, *coa_c* coenzyme A, *cptrc_c* N-carbamoylputrescine, *ddhkACPact_c* 6-deoxydihydrokalafungin, *dhkACPact_c* dihydrokalafungin, *dhkdhqn_c* dihydrokalafungin dihydroquinone form, *ephxcoa_c* *trans*-2,3-epoxyhexanoyl-CoA, *fmn_c *flavin mononucelotide, *fmnh2_c* reduced FMN, *fru_c* D-fructose, *fum_c* fumarate, *glc__D_c* d-glucose, *glcr_c* d-glucarate, *gly_c* glycine, *glyc__R_c* (R)-glycerate, *h2o_c* H_2_O, *h_c H* ^+^ , *h_e H* ^+^ , *hdhk_c* hydroxylated dihydrokalafungin, *hom__L_c* l-homoserine, *hpyr_c* hydroxypyruvate, *hx2coa_c* *trans*-hex-2-enoyl-CoA, *lipoamp_c* lipoyl-AMP, *lipoate_c* lipoate, *lipopb_c* protein N6-(lipoyl)lysine,* malcoa_c* malonyl-CoA,* mmcoa__S_c* (S)-methylmalonyl-CoA, *mql9_c* menaquinol 9, *mqn9_c* menaquinone 9, *nad_c* nicotinamide adenine dinucleotide, *nadh_c* nicotinamide adenine dinucleotide—reduced, *nadp_c* nicotinamide adenine dinucleotide phosphate, *nadph_c* nicotinamide adenine dinucleotide phosphate—reduced, *nh4_c *ammonium, *no_c* nitric oxide, *no3_c* nitrate, *o2_c* O_2_, *octa_c* octanoate (n-C8:0), *octapb_c* protein N6-(octanoyl)lysine,* oxadpcoa_*c3-oxoadipyl-CoA,*pac_c* phenylacetic acid, *phaccoa_c* phenylacetyl-CoA, *ppcoa_c* propanoyl-CoA,* ppi_c* diphosphate, *ptrc_c *putrescine, *salc_c* salicylate, *so3_c* sulfite, *succ_c* succinate, *succoa_c* succinyl-CoA, *taur_c* taurine, *urea_c* urea, *xyl__D_c* d-xylose, *xylu__D_c*D-xylulose.

To gain insights into the metabolic effects of losing these 30 reactions, we first conducted gene/reaction essentiality analysis for the two GEMs, SrapWT2040 and SrapUV2010 (Fig. 4b). We found that none of these 30 reactions were essential for the growth of the wild-type and the SMRK07 strain. Overall, SrapUV2010 showed a slightly greater number of essential genes and essential reactions than SrapWT2040: 189 versus 181 essential genes, and 513 versus 512 essential reactions (Fig. 4b). A possible reason for this observation is likely attributed to less metabolic robustness of SrapUV2010 as a result of losing the 30 reactions. For example, NAD kinase is encoded by two paralogous *nadK* genes in the wild-type, but, only one *nadK* gene remains in the SRMK07 strain. Therefore, this single *nadK* gene becomes essential in SrapUV2010 upon gene deletion in silico. Also, one additional essential reaction in SrapUV2010 corresponds to the reaction LIPOCT catalyzed by lipoyl(octanoyl) transferase. Likewise, LIPOCT is a non-essential reaction in the wild-type, but became essential as a result of deleting the reaction OCTNLL that is catalyzed by octanoate non-lipoylated apo domain ligase in ‘lipoate metabolism’. Both OCTNLL and LIPOCT contribute to the biosynthesis of lipoate, which is an essential cofactor for 2-oxoacid dehydrogenases and glycine cleavage system in central carbon metabolism^41^.

**Figure 5:**
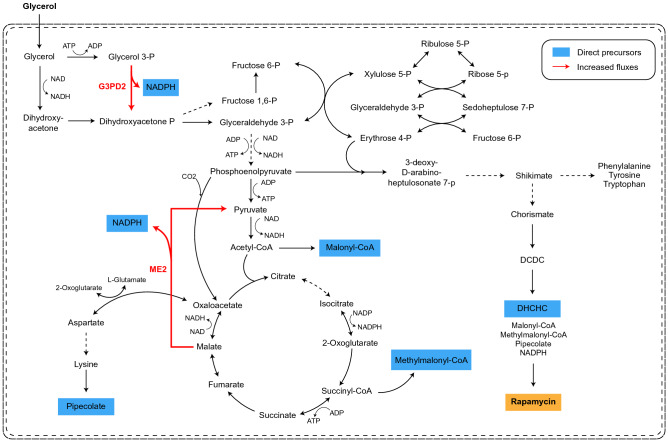
Prediction of intracellular metabolic flux distributions predicted using the genome-scale metabolic models (GEMs) of *S. rapamycinicus* NRRL 5491 (wild-type) and its mutant SRMK07. Intracellular metabolic flux distributions of the wild-type and the SRMK07 strain were predicted using parsimonious flux balance analysis (pFBA) of the GEMs. Metabolic fluxes predicted to be increased in the SRMK07 strain, compared to the wild-type, are marked with a red arrow. Direct precursors for rapamycin biosynthesis are shown in blue boxes. Dotted arrows indicate multiple reactions. Metabolite abbreviations are: *DCDC* 4,5-dihydroxycyclohexa-1,5-dienecarboxylic acid, *DHCHC* 4,5-dihydroxycyclohex-1-enecarboxylic acid.

Next, parsimonious flux balance analysis (pFBA) was implemented for SrapWT2040 and SrapUV2010 to gain insights into their intracellular flux distributions when producing rapamycin. The pFBA simulation revealed two reactions with greater flux values in SrapUV2010, ME2 (catalyzed by NADP-dependent malic enzyme) and G3PD2 (NADP-dependent glycerol-3-phosphate dehydrogenase) (Fig. 5). ME2 and G3PD2 both produce NADPH, which is a required cofactor for the biosynthesis of various secondary metabolites^42,43^; greater activities of these two corresponding enzymes could be another factor for the SRMK07 strain’s enhanced rapamycin production performance. According to a previous study, overexpressing *sco5261* for the ME2 reaction increased the production of a secondary metabolite actinorhodin in *S. coelicolor*^44^. Genes for these two reactions have never been targeted for the enhanced production of rapamycin, and thus, can be considered as overexpression targets for metabolic engineering of *S. rapamycinicus* in the future.

## Discussion

In this study, we conducted a comparative genomic analysis for *S. rapamycinicus* NRRL 5491 and its mutant strain SRMK07 that overproduces rapamycin. For this, both strains were subjected to WGS, which subsequently allowed the identification of large deletions at both end regions of the SRMK07 genome as well as the potentially duplicated region that covers the rapamycin BGC. The duplication of the rapamycin BGC as well as the deletion of the extremities of the chromosome that includes many BGCs are likely to have positive effects on the rapamycin biosynthesis. Obviously, duplicated rapamycin BGC would increase the dosage of rapamycin biosynthetic and regulatory genes, contributing to the enhanced biosynthesis of this molecule. Also, in the absence of multiple BGCs, precursors and energy used for the biosynthesis of the corresponding molecules may be redirected toward the rapamycin biosynthesis, further improving the rapamycin production. Core gene analysis was additionally conducted using ARTS to explain the SRMK07 strain’s normal growth despite the large genomic deletions. Finally, GEMs of the wild-type and the SRMK07 strain were reconstructed to examine these two strains’ metabolic differences.

This study suggests future research opportunities in metabolic engineering for the enhanced production of rapamycin and other secondary metabolites. Previous studies have shown the benefits of genome reduction, which reduces biological complexity, increases genome stability, and improves the production of secondary metabolites^45^. Relevant examples include *Streptomyces avermitilis* SUKA17 producing streptomycin and cephamycin^46^, *S. coelicolor* M1152 and M1154, both strains producing actinorhodin and chloramphenicol^47^, and *Streptomyces* sp. FR-008 LQ3 as a chassis for heterologous expression of biosynthetic genes for secondary metabolites^48^. Therefore, the SRMK07 strain can also be considered as a promising platform to construct a novel superhost for further enhanced production of rapamycin and other secondary metabolites^49,50^. Also, in addition to the two reactions ME2 (catalyzed by NADP-dependent malic enzyme) and G3PD2 (NADP-dependent glycerol-3-phosphate dehydrogenase) that can be considered as overexpression targets, further gene manipulation targets can be systematically predicted via comprehensive simulation studies using the GEMs reconstructed. Finally, additional omics analyses, for example transcriptome and metabolome, in combination with genomic and metabolic network analyses would provide more comprehensive phenotypic profiles of the wild-type and the SRMK07 strain.

## Conclusions

Comparative genomic analysis conducted in this study generated biological clues that could explain the enhanced rapamycin production performance of the *S. rapamycinicus* SRMK07 strain that was previously generated via random mutagenesis. The genomic and computational approaches undertaken in this study suggest gene manipulation targets to further enhance the production of rapamycin that can be experimentally tested through metabolic engineering. The approaches in this study can also be considered for analyzing other mutant strains generated from random mutagenesis.

## Methods

### Strains

*S. rapamycinicus* NRRL 5491, the wild-type, and its rapamycin overproducing mutant SRMK07 were used in this study. The SRMK07 strain was previously generated via UV-based random mutagenesis^24^. The wild-type spores were resuspended in saline buffer (0.85% NaCl and 0.1% Tween 90) to dilute the concentration to about 10^8^ /mL. Next, 100 µL of the diluted spores were spread evenly on a M1 plate (2.5 g/L corn steep powder, 3 g/L yeast extract, 3 g/L CaCO_3_, 0.3 g/L FeSO_4_, 10 g/L wheat starch, and 20 g/L agar), and exposed to UV for 60 s. UV conditions were set to be 254 nm wavelength, 40 W, and 25–30 cm distance from the spore suspension to achieve a 99% killing rate. The UV-treated spores were incubated at 28 °C for 7–10 days using agar plates containing 2 g/L rapamycin, which allowed the screening of rapamycin-resistant strains. The SRMK07 strain used in this study was obtained by measuring rapamycin from the resistant strains through liquid culture in a 250 mL flask.

### Cultivation conditions

Seed cultures of the two strains were incubated in GYM medium for 3 days, and transferred to a main cultivation medium. Flask cultivations were carried out for 14 days at 28 °C and 250 rpm (Fig. 1a,b). GYM medium contains: 4 g/L glucose, 4 g/L yeast extract, and 10 g/L malt extract. The main medium used in flask cultivations was adopted from Yun et al.^7^ with a slight modification. The main medium contains: 10 g/L M100, 50 g/L glycerol, 10 g/L cottonseed meal, 10 g/L soybean meal, 6.5 g/L yeast extract, 5 g/L (NH_4_)_2_SO_4_, 20 g/L L-lysine, 4 g/L L-tyrosine, 0.7 g/L KH_2_PO_4_, 1.14 g/L K_2_HPO_4_, 5 g/L NaCl, 0.05 g/L FeSO_4_·7H_2_O, and 42.6 g/L MES.

The two strains were also cultured on solid media in order to find differences in their growth phenotypes with focus on morphology and sporulation. ISP2 plate (4 g/L glucose, 4 g/L yeast extract, 10 g/L malt extract, and 20 g/L agar) was used to compare general growth characteristics of the two strains. M1 plate was used to examine sporulation of the two strains. The two strains were grown on the solid media for 7 days.

### Measurement of rapamycin concentration and packed mycelium volume (PMV)

During the flask cultivations, culture broth was sampled at 500 μL, and used for the measurement of rapamycin concentration. For this, culture broth was mixed with methanol in a 1:1 ratio, and vortexed for 30 min. The mixed solutions were subsequently centrifuged, and the supernatants were collected for the analysis using Waters 2695 (Waters, Milford, MA) high-performance liquid chromatography (HPLC) equipped with Agilent Eclipse XDB-C18 column (Agilent Technologies, Santa Clara, CA) and Waters 2487 detector (Waters, Milford, MA). In this HPLC analysis, water and acetonitrile were used as a mobile phase with ratio varied from 80:20 (v/v) to 20:80 (v/v) at a 1 mL/min flow rate, and 277 nm wavelength was used for the detector. Detected peaks were compared with a peak of the standard rapamycin compound (Sigma-Aldrich, St. Louis, MO) to measure the rapamycin concentration.

Packed mycelium volume (PMV) was used to estimate the growth of the NRRL 5491 and SRMK07 strains (Fig. 1b) because it was difficult to measure optical density or dry cell weight (DCW) from the insoluble media used for the rapamycin production^51,52^. For this, the collected culture broth (each 5 mL) was centrifuged at 3000 × *g* for 20 min. PMV was expressed as a percentage (%) by dividing PMV by the sample volume (5 mL).

### Whole genome sequencing

For WGS, the wild-type and SRMK07 strain were incubated in tryptic soy broth (TSB) medium (17 g/L tryptone, 3 g/L soytone, 2.5 g/L glucose, 5 g/L NaCl, and 2.5 g/L K_2_HPO_4_) for 3–4 days, and gDNA samples of the two strains were extracted using Wizard Genomic DNA Purification Kit (Promega, Madison, WI). Next, WGS and genome annotation of the two strains were conducted at DNA Link, Inc. (Seoul, Korea) by using the PacBio (Pacific Biosciences, Menlo Park, CA) and Illumina (Illumina Inc., San Diego, CA) platforms together. To increase the genome sequence quality, the genome correction method suggested by Lee et al.^53^ was used; if more than 80% of Illumina reads for a specific genomic site conflict with the PacBio results, these sequences were substituted according to the Illumina results using CLC Genomics Workbench version 6.5.1 (CLC bio, Aarhus, Denmark).

### Real-time PCR (qPCR) for verifying the potentially duplicated region in the SRMK07 strain’s genome

To verify the potentially duplicated region in the SRMK07 strain’s genome, five genes known to exist as a single copy in more than 100 *Streptomyces* species were selected based on OrthoDB^28^ (https://www.orthodb.org), and eight genes that represent the potentially duplicated region were selected based on our genome annotation results (Fig. 3). The qPCR experiments were conducted in accordance with the manufacturer’s protocol using gDNA of the two strains and SYBR Green PCR Master Mix (Thermo Fisher Scientific, Waltham, MA).

### Analysis of biosynthetic gene clusters (BGCs) and core genes

BGCs and core genes of the wild-type and SRMK07 strain were analyzed using antiSMASH version 5.0^27^ (http://antismash.secondarymetabolites.org) and ARTS (Antibiotic Resistant Target Seeker)^31^ version 2 (https://arts.ziemertlab.com), respectively. antiSMASH was implemented using the default options and ‘loose’ detection strictness. ARTS was also implemented with the default options with ‘Actinobacteria’ for ‘Reference set’.

### Generation of draft genome-scale metabolic models (GEMs)

The draft GEMs that represent the metabolism of the NRRL 5491 and SRMK07 strains were generated using a Python-based GEM reconstruction tool that was previously released as a feature of antiSMASH 3.0^54^. The Python-based GEM reconstruction tool requires protein sequences and their corresponding enzyme commission (EC) numbers for a target organism as well as a high-quality GEM of a biologically close organism in order to build a draft GEM. In this study, EC numbers for protein sequences from the wild-type and SRMK07 strain were predicted using DeepEC, a deep learning-based EC number prediction tool^55^. A high-quality GEM of *S. coelicolor*, iKS1317^56^, was used as a template GEM. The resulting draft GEMs were generated in Systems Biology Markup Language (SBML), and further model refinement and simulations were implemented using COBRApy^57^.

### Refinement of the draft GEMs

The draft GEM for the wild-type was first validated using the reported experimental growth data of *S. rapamycinicus* NRRL 5491 that involved 17 individual carbon sources and 19 individual nitrogen sources^58^ (Fig. 4c, Supplementary Tables S4, S5). Simulation of the draft GEM showed reasonably high accuracy, 78%, in comparison with the experimental growth data. Next, information on the rapamycin biosynthetic pathway, involving 14 condensation steps, a ring closure step, and post-polyketide synthase modification steps^22^, was added to the draft GEM. This rapamycin biosynthetic pathway was expressed as a single stoichiometric equation by implementing Biosynthetic Gene cluster Metabolic pathway Construction (BiGMeC), a pipeline that helps to create a metabolic pathway for a biosynthetic gene cluster encoding polyketides and non-ribosomal peptides^59^. Finally, MEMOTE (i.e., metabolic model tests)^60^ was implemented to evaluate the quality of the draft GEM. The same procedure was undertaken for the GEM representing the SRMK07 strain.

### Prediction of intracellular metabolic flux distributions using the GEMs

Parsimonious flux balance analysis (pFBA) was implemented to predict intracellular metabolic flux distributions because of its robust predictive power^61^. For pFBA of SrapWT2040, rapamycin production rate of 5 × 10^–4^ mmol/g DCW/h and glycerol uptake rate of 0.8 mmol/g DCW/h were provided as constraints based on a previous study^62^. For SrapUV2010, rapamycin production rate and glycerol uptake rate were set to 2.0 × 10^–3^ mmol/g DCW/h and 1.0 mmol/g DCW/h, respectively; rapamycin production rate and glycerol uptake rate were adopted from our cultivation experiments (Fig. 1) and Wang et al.^63^.

## Supplementary Information

Supplementary Information 1.

Supplementary Information 2.

Supplementary Information 3.

Supplementary Information 4.

## Data Availability

All the data generated or analyzed during this study, including genome-scale metabolic models (GEMs), are available as Supplementary Information files. Genome sequences of *S. rapamycinicus* NRRL 5491 and its mutant SMRK07 have been deposited in NCBI GenBank under accession numbers of CP085193 and CP085309.

## Abbreviations

BGC: Biosynthetic gene cluster
DCW: Dry cell weight
EC number: Enzyme commission number
gDNA: Genomic DNA
GEM: Genome-scale metabolic model
HPLC: High-performance liquid chromatography
NCBI: National Center for Biotechnology Information
pFBA: Parsimonious flux balanced analysis
PMV: Packed mycelium volume
qPCR: Real-time polymerase chain reaction
SBML: Systems Biology Markup Language
TIR: Terminal inverted repeat
TSB: Tryptic soy broth
UV: Ultraviolet
WGS: Whole genome sequencing
