# Supplementary material for: Comparative genomic analysis of Streptomyces rapamycinicus NRRL 5491 and its mutant overproducing rapamycin
Source: Sci Rep. 2022 Jun 18;12:10302. doi: 10.1038/s41598-022-14199-6 (PMC9206652; doi:10.1038/s41598-022-14199-6)
Supplement: Supplementary file 1 — Supplementary Information 1. [file 41598_2022_14199_MOESM1_ESM.pdf]

Supplementary Information

Comparative genomic analysis of *Streptomyces rapamycinicus* NRRL 5491 and its mutant overproducing rapamycin

Hee-Geun Jo[1,4], Joshua Julio Adidjaja[2,4], Do-Kyung Kim[1], Bu-Soo Park[1], Namil Lee[3], Byung-Kwan Cho[3], Hyun Uk Kim[2*] and Min-Kyu Oh[1*]

[1]Department of Chemical and Biological Engineering, Korea University, Seoul 02841, Republic of Korea

[2]Department of Chemical and Biomolecular Engineering (BK21 four), Korea Advanced Institute of Science and Technology (KAIST), Daejeon 34141, Republic of Korea

[3]Department of Biological Sciences, Korea Advanced Institute of Science and Technology (KAIST), Daejeon 34141, Republic of Korea

[4]Hee-Geun Jo and Joshua Julio Adidjaja contributed equally to this work

[*]email: ehukim@kaist.ac.kr; mkoh@korea.ac.kr

Supplementary Figures

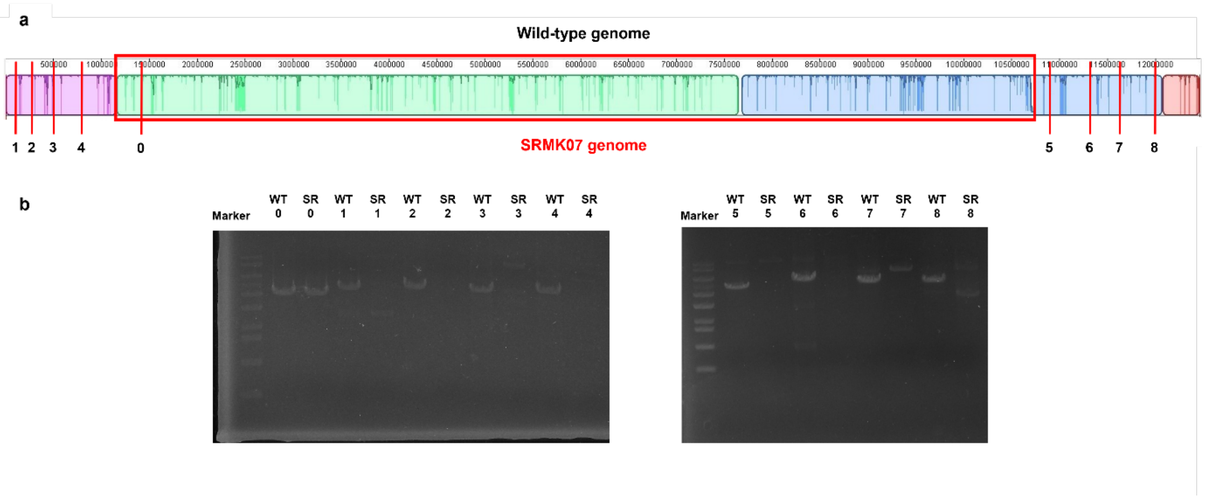

Supplementary Figure S1. PCR experiments to verify the potentially deleted regions in the SRMK07 strain's genome. (**a**) Location of primers (numbers '1' to '8') for the PCR experiments in the SRMK07 strain's genome. A red rectangle in the middle indicates the region where the genomes of the wild-type and the SRMK07 strain overlap. The region '0' has consistent sequences in both genomes of NRRL 5491 and SRMK07 as a positive control. The primer sequences are available in Supplementary Table S1, and each set of primers was designed to generate a product size of 4 to 5 kb. (**b**) Results of the PCR experiments. The target bands were observed only from the wild-type gDNA ('WT'). 'SR' indicates the results from the SRMK07 strain's gDNA.

Supplementary Tables

Supplementary Table S1. Primer sequences for PCR experiments to verify the potentially deleted regions in the SRMK07 genome.

Primer name[a]	Sequence
Set0_Fwd	CCTCCGCGTATCTCAAGAAG
Set0_Rev	GAGGCGGTGCTCTATCTCAC
Set1_Fwd	GCTCCCACCAGAACCTCTC
Set1_Rev	TCCTCTGGACGAACTCAACC
Set2_Fwd	CTGTCCGTCTTCCCCTACAC
Set2_Rev	GGGGCTCTCGTAGATGTCTG
Set3_Fwd	ACATCGACTTCACCGATTCC
Set3_Rev	ACCGTCACTTCTCCGTCAAC
Set4_Fwd	GGTCATGCTCACTCCATCCT
Set4_Rev	CACACTGCGCTTGTACTCGT
Set5_Fwd	CAATTCCCAGGATTGACTGG
Set5_Rev	CGCACAGCTCCAGGTAGTAA
Set6_Fwd	CTCATCGACCTCTCCGTACC
Set6_Rev	GGCTCGATCTTCGTGTTCTC
Set7_Fwd	GGCGCTTGTAGACACTCACA
Set7_Rev	TCTACCACCCCAACTTCGAG
Set8_Fwd	GGCAACAACAAGTGGAAGGT
Set8_Rev	GCGAGAACAAGAGGATCGAC

[a] Suffixes ‘Fwd’ and ‘Rev’ indicate forward and reverse primers for each gene, respectively.

Supplementary Table S2. Primer sequences for qPCR experiments to verify the potentially duplicated region in the SRMK07 genome.

Primer name[a]	Sequence
R1_Fwd	CGCAGAAGGAAGACATCGTA
R1_Rev	CGACGAGGACGAGGAGATAC
R2_Fwd	GTACAAGCCGATCGAGAAGG
R2_Rev	CTTCACGCACACCACCTG
R3_Fwd	ACTTCCTGGCCTTCCTCAAT
R3_Rev	TGTAGATGTCCTGCCATTCG
R4_Fwd	GGTACACCATCCCCATTCAC
R4_Rev	CTGCTCCATGCCTTCTTCTC
R5_Fwd	GGCTCTACGAGCTGATCTGG
R5_Rev	GATCTTGACGGTGACCGAGT
T1_Fwd	ATGTCGTGCTGAACTCGTTG
T1_Rev	GATCACGGATGTCGGTCTTG
T2_Fwd	ATCTGCTGGAGGAACTGTCG
T2_Rev	CTCCCACTCGTAGGAGAACC
T3_Fwd	ATGCAGACCAAGGTTCTGTG
T3_Rev	CTGATCAGCCGATCCATCAT
T4_Fwd	GATGGGAGCTGTGGATCG
T4_Rev	CGTGCATCTCGAAGTACGC
T5_Fwd	GTGTTCGTGAACGTGGACAG
T5_Rev	GACGGCCCTTCTCTATGAGG
T6_Fwd	GCCCACTACCTGCACACC
T6_Rev	GGTCGACCACCAGCAGTC
T7_Fwd	TCGATGTCCTGGTCAACAAC
T7_Rev	ACTCGAAATTGAGGGCGAAC
T8_Fwd	GAGATGGCCTCCCGCTAT
T8_Rev	GCATGGTGATGTCCTGTTTG

[a] Suffixes ‘Fwd’ and ‘Rev’ indicate forward and reverse primers for each gene, respectively.

Supplementary Table S3. Futalosine biosynthetic genes in *Streptomyces coelicolor* and their corresponding locations in the genomes of *Streptomyces rapamycinicus* NRRL 5491 and SRMK07.

Streptomyces coelicolor	***Streptomyces rapamycinicus NRRL 5491***	***Streptomyces rapamycinicus* SRMK07**
sco4506 (*mqnA*)	5,444,627 - 5,445,445 (NJMEEBPL_04454)	4,269,599 - 4,270,417 (JDDNEILB_04407)
sco4327 (*mqnB*)	6,736,979 - 6,737,806 (NJMEEBPL_05641)	5,561,924 - 5,562,751 (JDDNEILB_03218)
sco4550 (*mqnC*)	5,441,425 - 5,442,624 (NJMEEBPL_04452)	4,266,397 - 4,267,596 (JDDNEILB_04409)
sco4326 (*mqnD*)	6,736,092 - 6,736,982 (NJMEEBPL_05640)	5,561,037 - 5,561,927 (JDDNEILB_03219)
sco4556	5,440,229 - 5,440,876 (NJMEEBPL_04450)	4,265,201 - 4,265,848 (JDDNEILB_04411)
sco4491	5,467,321 - 5,468,244 (NJMEEBPL_04480)	4,292,293 - 4,293,216 (JDDNEILB_04381)
sco4490	5,468,241 - 5,469,692 (NJMEEBPL_04481)	4,293,213 - 4,294,664 (JDDNEILB_04380)
sco4492	5,466,565 - 5,467,296 (NJMEEBPL_04479)	4,291,537 - 4,292,268 (JDDNEILB_04382)

Supplementary Table S4. Predicted growth of *Streptomyces rapamycinicus* NRRL 5491 using its genome-scale metabolic model SrapWT2040 under different carbon sources.

**Carbon source**	**Exchange reaction ID**	**Experimental phenotype[a]**	**Predicted phenotype**
D-Fructose	EX_fru_e	+	+
D-Glucose	EX_glc__D_e	+	+
Glycerol	EX_glyc_e	+	+
Maltose	EX_malt_e	+	+
D-Mannitol	EX_mnl_e	+	+
D-Mannose	EX_man_e	+	+
Raffinose	EX_raffin_e	+	+
L-Rhamnose	EX_rmn_e	+	+
D-Ribose	EX_rib__D_e	+	+
Trehalose	EX_tre_e	+	+
D-Xylose	EX_xyl__D_e	+	+
L-Arabinose	EX_arab__L_e	+	+
Inositol	EX_inost_e	+	-
α-D-Lactose	EX_lcts_e	+	+
Salicin	EX_salcn_e	+	+
Glucosamine	EX_gam_e	-	-
D-Sorbitol	EX_sbt__D_e	-	+

[a] Data taken from Kumar and Goodfellow [1].

Supplementary Table S5. Predicted growth of *Streptomyces rapamycinicus* NRRL 5491 using its genome-scale metabolic model SrapWT2040 under different nitrogen sources.

Nitrogen source	Exchange reaction ID	Experimental phenotype[a]	Predicted phenotype
L-Arginine	EX_arg__L_e	+	+
L-Asparagine	EX_asn__L_e	+	+
L-Glutamic acid	EX_glu__L_e	+	+
L-Histidine	EX_his__L_e	+	+
L-isoleucine	EX_ile__L_e	+	+
L-Ornithine	EX_orn_e	+	+
L-Proline	EX_pro__L_e	+	+
L-Threonine	EX_thr__L_e	+	+
α-Alanine	EX_ala__D_e	+	+
L-Alanine	EX_ala__L_e	+	+
L-Glycine	EX_gly_e	-	+
DL-Methionine	EX_met__L_e	+	-
L-Phenylalanine	EX_phe__L_e	+	+
L-Serine	EX_ser__L_e	+	+
L-tryptophan	EX_trp__L_e	-	+
L-Valine	EX_val__L_e	+	+
L-Aminobutyric acid	EX_4abut_e	-	+
DL-Aspartic acid	EX_asp__L_e	-	+
L-Leucine	EX_leu__L_e	-	+

[a] Data taken from Kumar and Goodfellow [1].

Supplementary Data

Supplementary Data S1. Genomic information of *Streptomyces rapamycinicus* NRRL 5491 and its mutant, SRMK07, overproducing rapamycin.

Supplementary Data S2. Core genes of *Streptomyces rapamycinicus* NRRL 5491 and its mutant, SRMK07, overproducing rapamycin.

Supplementary Data S3. Genome-scale metabolic model of *Streptomyces rapamycinicus* NRRL 5491, SrapWT2040, in the Systems Biology Markup Language (SBML) format.

Supplementary Data S4. Genome-scale metabolic model of *Streptomyces rapamycinicus* SRMK07, SrapUV2010, in the Systems Biology Markup Language (SBML) format.

Supplementary References

1 Kumar, Y. & Goodfellow, M. Five new members of the *Streptomyces violaceusniger* 16S rRNA gene clade: *Streptomyces castelarensis* sp. nov., comb. nov., *Streptomyces himastatinicus* sp. nov., *Streptomyces mordarskii* sp. nov., *Streptomyces rapamycinicus* sp. nov. and *Streptomyces ruanii* sp. nov. *Int J Syst Evol Microbiol* **58**, 1369-1378. https://doi.org/10.1099/ijs.0.65408-0 (2008).
